# Insulin Resistance in Peripheral Tissues and the Brain: A Tale of Two Sites

**DOI:** 10.3390/biomedicines10071582

**Published:** 2022-07-02

**Authors:** Elizabeth M. Rhea, William A. Banks, Jacob Raber

**Affiliations:** 1 Department of Medicine, Division of Gerontology and Geriatric Medicine, University of Washington, Seattle, WA 98195, USA; meredime@uw.edu (E.M.R.); wabanks1@uw.edu (W.A.B.); 2 Geriatric Research Education and Clinical Center, Veterans Affairs Puget Sound Health Care System, Seattle, WA 98108, USA; 3 Department of Behavioral Neuroscience, Oregon Health & Science University, Portland, OR 97239, USA; 4 Departments of Neurology and Radiation Medicine, Division of Neuroscience, ONPRC, Oregon Health & Science University, Portland, OR 97239, USA

**Keywords:** insulin resistance, central nervous system, apolipoprotein E

## Abstract

The concept of insulin resistance has been around since a few decades after the discovery of insulin itself. To allude to the classic Charles Dicken’s novel published 62 years before the discovery of insulin, in some ways, this is the best of times, as the concept of insulin resistance has expanded to include the brain, with the realization that insulin has a life beyond the regulation of glucose. In other ways, it is the worst of times as insulin resistance is implicated in devastating diseases, including diabetes mellitus, obesity, and Alzheimer’s disease (AD) that affect the brain. Peripheral insulin resistance affects nearly a quarter of the United States population in adults over age 20. More recently, it has been implicated in AD, with the degree of brain insulin resistance correlating with cognitive decline. This has led to the investigation of brain or central nervous system (CNS) insulin resistance and the question of the relation between CNS and peripheral insulin resistance. While both may involve dysregulated insulin signaling, the two conditions are not identical and not always interlinked. In this review, we compare and contrast the similarities and differences between peripheral and CNS insulin resistance. We also discuss how an apolipoprotein involved in insulin signaling and related to AD, apolipoprotein E (apoE), has distinct pools in the periphery and CNS and can indirectly affect each system. As these systems are both separated but also linked via the blood–brain barrier (BBB), we discuss the role of the BBB in mediating some of the connections between insulin resistance in the brain and in the peripheral tissues.

## 1. Introduction

The idea of insulin resistance was firmly established among clinicians by the early 1940′s who had observed that there were rare patients who required large doses of insulin to control blood sugar [1,2,3]. At about the same time, the first example of resistance to an endogenous hormone was reported when patients who had the phenotype associated with low blood levels of parathyroid hormone were found to have high levels instead [4]. This particular case of hormone resistance, pseudohypoparathyroidism, was eventually found to be due to the occurrence of low levels of receptors for parathyroid hormone. Over the subsequent decades, the concept of hormone resistance expanded in two ways [5]. One way was that other hormones were found to have resistance syndromes. The second way was that causes of hormone resistance in addition to receptor deficiencies were found. The causes of hormone resistance include production of inactive/underactive hormone, antibodies that block hormone/receptor interactions, decreased hormone receptor number, decreased post receptor signaling, and downstream unresponsiveness. Interestingly, many of the causes of hormone resistance listed above, such as antibodies directed against the hormone or its receptor, were first described for insulin. When a hormone depends on transport across a barrier, including the blood–brain barrier (BBB), to reach its site of action, then defective transport can also result in hormone resistance. In all of these examples, elevated blood levels of hormone occur in the face of deficient hormonal activity.

Insulin resistance in mammals is classically defined as an inadequate response of the insulin-sensitive tissues, that is, those tissues (skeletal muscle, liver, and fat) whose glucose handling is regulated by insulin, resulting in the maintenance of euglycemia. With the occurrence of insulin resistance, ever higher levels of insulin are needed to maintain euglycemia until the limits of the system are reached, resulting in hyperglycemia. Yet, insulin signaling is present in nearly every tissue, including the CNS. While these tissues do not participate in the negative feedback loop that locks together the levels of insulin and glucose, they are still dependent on the pancreas for their insulin. Although the “insulin-insensitive” tissues have glucose transporters that are not regulated by insulin, insulin controls various aspects of their physiology that relate to metabolic and mitogenic functions [6]. In this sense, most, if not all, tissues are “insulin sensitive”, even those whose glucose transporters are not regulated by insulin. It is now well established that other factors besides glucose that can stimulate insulin release, activity and metabolism, such as fructose, free fatty acids, and amino acids [7]. However, it is glucose that is the primary determinant of insulin secretion and it is the glucose response by which insulin sensitivity and resistance is mainly measured.

Insulin resistance is commonly defined by absent or inadequate response to insulin. Insulin resistance precedes many common diseases associated with this disorder, including type 2 diabetes. While it has not been directly tested, it is postulated that CNS insulin resistance precedes cognitive decline, as the degree of CNS insulin resistance correlates with cognitive decline [8]. When referring to CNS insulin resistance throughout this review, the primary component involves the brain. Spinal cord insulin resistance has not been investigated in great detail in cognitive decline. Deregulated nutrient sensing, to which insulin resistance can be considered a component, is a part of aging and is considered one of the nine original Hallmarks of Aging [9], though this concept is likely attributed in part to peripheral insulin signaling and the insulin-like growth factor 1 (IGF-1) system. The insulin/IGF-1 system is a well-known nutrient sensing pathway that is also implicated in lifespan extension [10]. The relation of deregulated nutrient sensing along a beneficial-deleterious spectrum to that of CNS insulin resistance and its relation to cognition is not well explored. 

The insulin signaling pathway is one of the most conserved aging-controlling pathways in evolution [9]. However, insulin as a hormone is synthesized exclusively by the pancreas in most vertebrates and having primarily a role in glucose regulation is a relatively “new” evolutionary innovation. In other vertebrates and invertebrates, insulin and ancestral insulins are secreted by many tissues and have both metabolic and mitogenic roles [6]. Insulin signaling is present in virtually every cell throughout the mammalian body [11], but the biological outcome is dependent on the cell type and is largely not related to glucose transport. There are many potential points where this signaling pathway can be dysregulated in multiple tissues. Peripheral insulin resistance is very prevalent among the United States (US) population, occurring in approximately 24% of adults older than 20 years [12], with this number increasing to 40% in those over 50 years [13]. CNS insulin resistance also occurs in many different groups: in individuals with peripheral insulin resistance, independently in aged individuals, in those with Alzheimer’s disease (AD) [8], or intertwined in individuals with AD and peripheral insulin resistance. This latter group, those with both AD and peripheral insulin resistance, is made up of approximately 35% of patients with AD [14]. 

In this review, we will briefly introduce the insulin receptor signaling pathway, the similarities and differences between the etiology of peripheral and CNS insulin resistance, the testing and determining of insulin resistance, and the consequences of these two conditions, focusing on the impact of aging and age-related cognitive decline. As apolipoprotein E (apoE) is linked to insulin signaling and age-related cognitive decline [15], we will also explore peripheral versus central action of the apoE isoforms. Lastly, we highlight recent data obtained on the impact of insulin resistance in the development of COVID-19 and the related cognitive injury.

### 1.1. Periphery versus CNS: Separate Entities Connected by a Regulated Interface

**Figure 1:**
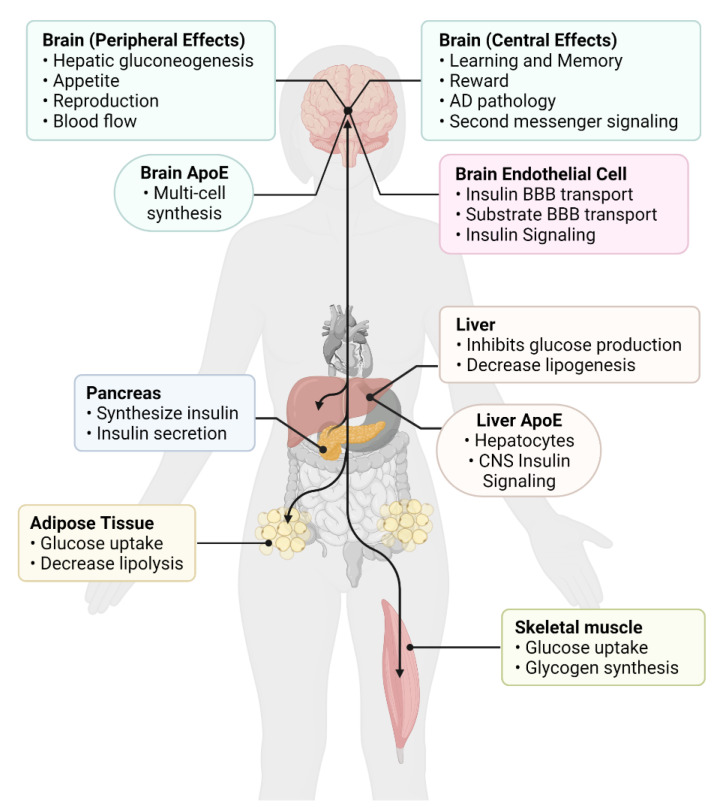
Overview of peripheral and central insulin actions. Insulin action primarily originates from the pancreas, the site of insulin production, and can act on many target tissues (black arrows). In the periphery, insulin has actions in all tissues, but predominantly affects major metabolic tissues, such as the adipose tissue, liver, and skeletal muscle. Insulin crosses the blood–brain barrier (BBB) to act within the central nervous system (CNS) to regulate both direct effects within the brain as well as elicit indirect signaling events back to the periphery. Insulin can also signal at the brain endothelial cell to regulate BBB function. On the other hand, apolipoprotein E (apoE) has distinct pools (peripheral and central), as apoE does not cross the BBB, with liver and brain producing the majority of these apoE pools. There is still crosstalk between these two pools as peripheral apoE can still readily affect central apoE, and vice versa, through indirect actions (i.e., via the gut microbiome and gut-liver-brain axis). Created with Biorender.com software.

We will discuss the similarities, differences, and associations between peripheral and CNS insulin resistance, touching on the role of insulin action in each of these systems, which are comprised of multiple organs or multiple contributing cell types (Figure 1). The definition of insulin resistance at peripheral tissues would seem easy to identify: whenever more than “normal” levels of insulin are needed to maintain blood glucose levels, insulin resistance is the presumptive cause. The blood levels of insulin and glucose are locked together in a negative feedback loop. When glucose levels rise under physiologic conditions, more insulin is secreted to bring blood glucose levels back into the normal range, at which point the stimulus for insulin secretion is abated and insulin secretion and insulin blood levels return to normal. When receptor resistance occurs, insulin sensitive tissues require more insulin than normal to take up glucose, resulting in a rise in insulin levels to the level needed to maintain euglycemia. When receptor resistance exceeds the capacity of the pancreas to secrete enough insulin to compensate for this resistance, hyperglycemia occurs. Thus, the term insulin resistance includes conditions with normal blood glucose levels (hyperinsulinemic euglycemia) as well as those with elevated glucose levels (hyperinsulinemic hyperglycemia). 

At first glance, then, defining CNS insulin resistance would seem straightforward: it would simply be an extension of the concepts of peripheral insulin resistance. However, partly because the CNS is its own sphere and partly because early assumptions have since been shown to not be true in all circumstances, the concept of CNS insulin resistance is under revision. In a later section, we consider the definition and some of the mechanisms of CNS insulin resistance, but in this section, we consider some of the erroneous assumptions that have complicated the field of CNS insulin resistance.

The first assumption that has not held is that CNS insulin resistance occurs as an extension of or in tandem with insulin resistance at peripheral tissues; in other words, hormonal resistance occurs simultaneously in all tissues, both peripheral and CNS. Therefore, since peripheral insulin resistance is associated with AD [16], there would be a CNS insulin resistance component that would also be associated with AD. This logic was reinforced by the findings that many risk factors for insulin resistance were also risk factors for AD, including obesity, hyperlipidemia, and inflammation [17]. Additionally, either diabetes mellitus [8] or a deficiency in CNS insulin action [8] have been associated with AD. While the inferences of the assumption have led to important discoveries, the assumption itself, that insulin resistance in the periphery and CNS occur simultaneously, is not always correct, as discussed next.

That the presence of peripheral insulin resistance does not infer the presence of CNS insulin resistance was shown in the landmark study of Talbot et al. [8]. This study showed that insulin receptor resistance in brain occurred in the majority of subjects with AD, whereas diabetes mellitus was absent in most. This indicted that CNS insulin resistance was present in the absence of peripheral insulin resistance. That receptor resistance can occur in some tissues but not others is a well-established feature of hormonal resistance. A classic example of this is provided by resistance to thyroid hormone [18]. Resistance to the thyroid hormone thyroxine can occur at the pituitary, at the hypothalamus, or at peripheral tissues such as gut, muscle, and heart. In the case of pituitary resistance and hypothalamic resistance, thyroxine levels are elevated in blood, whereas when resistance does not include these tissues, thyroxine levels are normal. This is because the pituitary and hypothalamus are part of the negative feedback loop of thyroid production, whereas tissues such as the gut, muscle, and heart are not part of that negative feedback loop. In the Allan–Herndon–Dudley syndrome, thyroid hormones are not transported across the BBB because of a defect in the thyroid hormone transporter [19]. In this case, the brain is in a hypothyroid state and unresponsive to thyroid replacement; that is, resistant to peripheral thyroid hormones.

Another reason that CNS insulin resistance is a confusing concept is that what actually constitutes CNS insulin resistance did not arise directly from the concepts elucidated for peripheral hormone resistance. It arose from the idea that increasing CNS insulin could improve cognition in AD [20]. However, this could be caused by a deficiency of insulin action within the CNS and deficiency in action could be caused by low levels of insulin in the CNS, as well as resistance to insulin at the receptor. Because CNS receptors do not participate in the classic negative feedback between glucose and insulin levels, blood insulin levels are largely unaffected by a deficient insulin activity in the CNS. This makes testing CNS insulin resistance difficult in living subjects.

A summary of the above is that CNS insulin resistance is a separate entity from that of peripheral insulin resistance. Although both can contribute to cognitive decline in general and contribute to AD, they likely do so through different pathways. CNS insulin resistance, as discussed further below, can include low levels of CNS insulin as well as resistance at the receptor level and therefore would be better described as a deficiency in CNS insulin action.

### 1.2. Peripheral versus CNS apoE Pools

ApoE synthesis occurs both in the periphery and the CNS, with the liver and the brain predominating in this synthesis [21]. Peripheral and CNS pools are separate, as apoE does not cross the BBB, best shown following liver transplantations [22]. In mice, only a single isoform of apoE is expressed, whereas in humans there are three major isoforms: the dominant E3, E4 (thought to promote AD), and E2 (thought to protect against AD) [23]. Plasma apoE is mainly derived from the liver [24] but apoE can also be synthesized in the spleen, kidney, lungs, adrenal gland, muscles, peripheral nervous system, and monocytes and macrophages [25]. Other tissues generating modulators important for regulating insulin sensitivity, including steroidogenic tissues [26], express apoE as well. For example, in the adrenal gland, most apoE is present in the cortical cells where glucocorticoids are generated [27,28]. In the adrenal gland, apoE mRNA is inversely related to steroidogenesis; when apoE mRNA in the adrenal gland increases, steroidogenesis is reduced and when apoE mRNA in the adrenal gland is decreased, steroidogenesis is increased [27,28]. Mice lacking apoE show an age-dependent increase in basal and restraint stress-induced plasma corticosterone levels associated with age-dependent changes in metabolism [29]. 

Although lack of BBB transport keeps the peripheral and CNS pools of apoE separated, this does not mean that peripheral apoE or the deficiency of peripheral apoE does not affect the CNS. Strikingly, mice lacking murine apoE and mice lacking murine apoE but expressing human apoE3 or apoE4 in brain show a similar increase in plasma corticosterone following restraint stress [29], in agreement with the inverse relationship between apoE mRNA in the adrenal gland steroidogenesis [27]. Consistent with peripheral apoE affecting the CNS, plasma apoE levels are positively correlated with severity of post-traumatic stress disorder (PTSD), a disorder afflicting the CNS [30]. There are also direct effects of apoE in brain that do not require peripheral apoE. For example, mice deficient in murine apoE but expressing human apoE3 in the CNS did not have age-dependent neuropathology in the hippocampus or cortex, while those without murine apoE but expressing human apoE4 in the CNS did [31]. By peripheral and central effects on the vasculature, central apoE can affect the periphery and peripheral apoE can affect the brain [32]. Increased efforts are warranted to study the effects of brain-derived apoE on the periphery.

The effects of peripheral apoE on the CNS might be related to the apoE isoform and/or the apoE levels. In human apoE targeted replacement mice exposed to a chronic variable stress paradigm, plasma apoE levels are higher in apoE2 than apoE3 and apoE wild-type mice [33]. Similarly, cerebellar apoE levels in apoE2 mice are higher than those in apoE3 and apoE4 mice [34]. There is a sex difference as well, with higher plasma apoE levels in females than males, likely driven by apoE2 expression [33]. Mouse apoE levels are also regulated by expression of one of its receptors, the low-density lipoprotein receptor (LDLR). Mice expressing apoE2 alongside human LDLR expression have higher plasma apoE levels compared to apoE3 or apoE4 mice [34]. More importantly, the presence of the human LDLR affect apoE levels in the brain, with higher apoE levels in the cortex, cerebellum, hippocampus, and amygdala driven by apoE2. 

Other effects of liver-derived apoE on the CNS are less clear. Deletion of apoE in the liver does not affect amyloid pathology in human apoE targeted replacement mice crossed with an AD mouse model (APP/PS1 mice) [35]. As noted above, in patients receiving liver transplants, the apoE isoform present in the plasma matches that of the donor while the apoE isoforms present in brain do not change [36]. However, increasing evidence supports effects of apoE in liver and plasma on the brain. For example, in brains of apoE4 mice with double-humanized liver and hematopoietic stem cells (FRGN mice), endogenous mouse apoE brain levels are lower and associated with alterations in markers of synaptic integrity, neuroinflammation, and insulin signaling compared to apoE2/E3 mice [37]. Specifically, liver apoE4 expression results in a decrease in insulin signaling in cortical neurons and is associated with the plasma apoE4 level. Consistent with this notion, neuropathology in the brain is seen in mice in which murine apoE in the liver is replaced by human apoE [38]. These effects of humanized and plasma apoE [22], might be mediated indirectly by both peripheral apoE levels and apoE isoforms. Low plasma apoE levels are associated with impaired cognition and brain pathology [38] and increased risk to develop dementia [39]. An increased ratio of plasma apoE4 to apoE3 isoform levels is associated with hippocampal glucose hypometabolism and neuropathology in different brain regions [40]. It is important to note that in *APOE* heterozygous individuals the plasma composition of the two apoE isoforms present is different from that in the cerebrospinal fluid (CSF) [41,42]. 

In CD11c^+^ microglia in the spinal cord, peripheral apoE is implicated in the resolution of neuropathic pain [43,44]. As apoE is also altered in disease-associated microglia, it illustrates that increases in apoE might either enhance inflammation and injury or help resolve it. As the development of insulin resistance, both in the periphery and the CNS, can involve inflammation, as will be discussed in more detail later, apoE pools could also be implicated in this process. This summary highlights the separate but interconnected pools of apoE in the periphery and CNS. Later, we will explore how apoE interacts with insulin signaling both in the periphery and in the CNS. Before that, we want to briefly describe the primary signaling events of insulin as mediated through the insulin receptor. 

## 2. Insulin Receptor and Signaling

**Figure 2:**
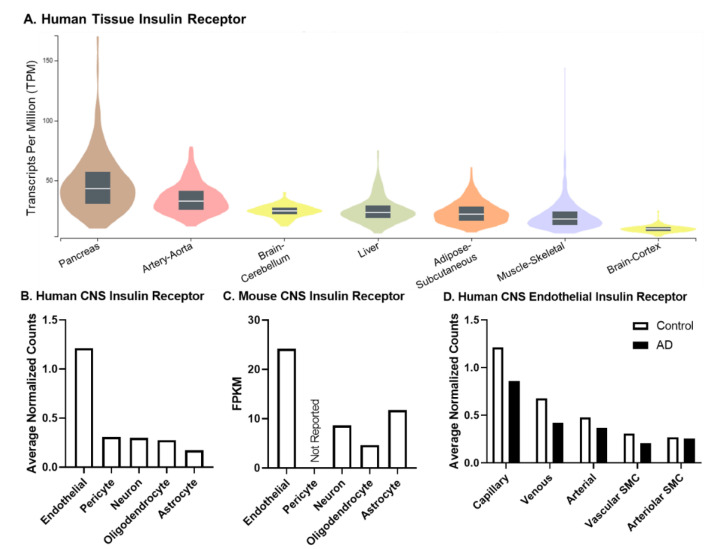
Expression (mRNA) of the insulin receptor (IR). Existing online, publicly available data resources were used to generate each sub-figure. (**A**) Expression of IR mRNA in human tissues was organized from the GTEx portal. Box plots are shown as the median, and 25th and 75th percentiles. The sample size range is 226–803. Outliers are not shown. The transcript data is based on the GTEx Analysis Release V8. (**B**) Human CNS IR data were extracted from the Human BBB Project [47] where the endothelial values shown are from capillary expression, pericytes are from solute transport pericytes, and astrocytes are from the cortex. Tissue samples were taken from post-mortem superior frontal cortex (*n* = 4) and hippocampus (*n* = 8–9) and pooled unless otherwise stated. (**C**) Mouse CNS IR data were extracted from the Brain RNA-Seq data base [48]. Briefly, two biological replicates were used to generate the database (one biological replicate consists of cells purified from 3–12 mouse cerebral cortices) and cell types were purified from different mice. Pericyte data were not shown due to the small contamination of astrocytes and endothelial cells. (**D**) Human CNS Endothelial IR data were also extracted from the Human BBB project [47]. Only the vascular cell types are shown and levels are compared between controls and AD samples. AD classification was based on a clinical diagnosis. All data were accessed on 4 April 2022.

The insulin receptor (IR) is a transmembrane protein that also acts as a critical tyrosine kinase important in intracellular signaling following ligand binding [45]. The IR is present in every tissue at different levels of expression. For example, the tissue responsible for the primary source of insulin, the pancreas, has the greatest level of IR mRNA (Figure 2A). IR mRNA expression in other tissues most commonly involved in insulin resistance is shown in Figure 2A. IR mRNA levels are higher in the cerebellum compared to the peripheral tissues most often studied for insulin resistance: the liver, skeletal muscle, and adipose tissue. Additionally, the aorta, a tissue rich in endothelial cells, has higher levels of IR mRNA compared to all these other tissues. With the technological advances of single-cell sequencing, recent studies have further investigated expression of IR mRNA in different cell types within the brain, including endothelial cells, pericytes, neurons, oligodendrocytes, and astrocytes (Figure 2B,C). The distribution of the IR is similar between the brain cell types in humans (Figure 2B) and mice (Figure 2C). Due to the brain vasculature acting as a critical interface between the periphery and the brain, IR mRNA expression can also be explored in the different brain vascular beds (Figure 2D). The majority of the IR is in the capillaries, where nutrient exchange most commonly takes place. Levels of the IR mRNA in these different vascular beds are decreased in AD (Figure 2D). However, it should be noted the IR mRNA values reported have been quite variable [46]. Therefore, follow up studies measuring protein levels are needed to fully assess the expression levels present in tissues, cell types, and even cerebrovascular beds. This is especially pertinent given that the single IR gene is spliced into two isoforms that are differentially expressed in the brain and during disease, as discussed next. 

The IR exists as two isoforms, an A and B isoform, differentiated by the splicing of exon 11, contributing to the addition of 12 amino acids (IR-B). Insulin binding affinity is similar between the two isoforms. However, IR-A and IR-B can form heterodimers with the insulin-like growth factor-1 receptor (IGF-1R), forming hybrid receptors, which have a lower affinity for insulin than homodimers [49]. IR-A has a greater affinity for IGF-1/2. The extra amino acids in IR-B hybrid receptors are thought to interfere with the affinity for ligands including insulin, IGF-1, and IGF-2. IR-A is predominantly viewed as important in development and IR-B is more critical for metabolism in adults. A recent preprint indicated that the expression of IR-A vs. IR-B shifts at the BBB in disease. IR-B is the predominant form found at the BBB in healthy conditions but there is a shift towards a higher IR-A/B ratio at the BBB in AD that correlates with cognitive score [50]. Perhaps a shift in the IR-A/B ratio at the BBB drives differences in the level of vascular binding for insulin in AD [51]. 

The canonical metabolic signaling pathway following activation of the IR is autophosphorylation of itself, followed by phosphorylation of the IR substrate 1/2 (IRS1/2), and protein kinase B (also known as Akt). Alternatively, IR activation can lead to activation of the MAPK pathway, which is considered the mitogenic signaling pathway. It has been proposed that down-regulation of the insulin signaling pathway is a defense mechanism to slow cell growth and metabolism under conditions of stress or damage [8]. If there are slower rates of cell growth, there are also slower rates of cellular damage. However, the balance of slowing cell growth must be weighed against the detrimental effects that can become deleterious or aggravate the aging process. Measuring the insulin-stimulated phosphorylation of these proteins can aid in measuring insulin resistance both in the periphery and the CNS. The measurement of IR and IRS1/2 phosphorylation is more specific to insulin activation, rather than Akt phosphorylation, which is a much broader downstream mediator. Using phosphorylated mediators of the IR as a readout for insulin resistance is more common in the CNS, requiring post-mortem tissue. This pathway and downstream signaling is critical in the development and perseverance of insulin resistance as will be discussed next.

## 3. Peripheral Insulin Resistance

**Figure 3:**
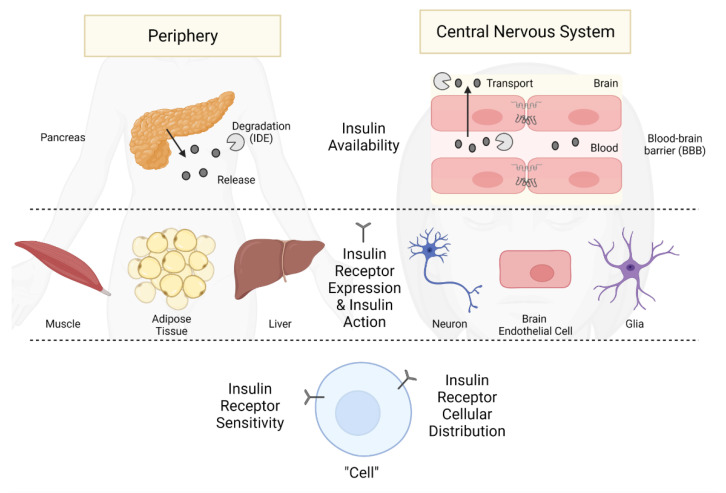
Points of Insulin Resistance. Insulin resistance can arise at many different levels that differ slightly whether it develops in the periphery or the CNS. First, the availability of insulin can lead to development of insulin resistance, with the pancreas regulating peripheral levels and the BBB primarily regulating CNS levels. Of course, degradation of insulin by the insulin degrading enzyme (IDE) can also contribute to availability in each of these pools. Second, the expression of the IR and ability to respond to insulin in tissues throughout the periphery or in different CNS cell types within the CNS can lead to insulin resistance. Lastly, at the cellular level, whether in the periphery or in the CNS, IR localization on the cell surface or the sensitivity of the IR to insulin can impact insulin resistance. Created with BioRender.com software.

Insulin is produced and released by beta cells within the pancreas in response to changes in glucose levels (Figure 3). The primary role of insulin in the periphery is to stimulate glucose handling by acting on the insulin-sensitive glucose transporters located predominantly on adipose tissue, muscle, and liver, allowing for the storage and generation of energy required by the body (Figure 1). While type 2 diabetes and obesity are most often associated with insulin resistance, there are many other diseases associated with insulin resistance, including genetic diseases discussed next and others such as chronic kidney disease [52]. Peripheral insulin resistance is often accompanied by high blood glucose levels and a state of positive energy balance, leading to obesity. Differentiating the CNS effects, particularly those related to cognition, due to either high blood glucose or high blood insulin levels can be difficult as both are often present in peripheral insulin resistance. This will be discussed in more detail later in this section.

There are very rare cases of mutations in the IR gene which can lead to insulin resistance, requiring 100-fold or more insulin than that required by a typical diabetic patient [53]. In this regard, there are various non-invasive insulin delivery mechanisms, including oral, transdermal, rectal, vaginal, ocular, and nasal involving smart insulin delivery systems (for a review, see [54]). However, there is an inverse dependence between IR expression at the cell membrane and insulin concentrations [55]. During hyperinsulinemic-resistant states in both rodents and humans, IR levels are decreased (reviewed in [55]). Not only does relocation of the IR affect the ability of the IR to respond to extracellular insulin, but often internalization of the receptor can lead to degradation [56]. Reductions in the level of the IR are not solely responsible for insulin resistance as mice expressing half of the normal level of the IR do not present with major metabolic abnormalities [57]. Hyperinsulinemia can also decrease the expression of downstream insulin signaling mediators IRS1 and IRS2, which have been linked to insulin resistance. Degradation and decreased synthesis can contribute to these decreased protein levels. The insulin degrading enzyme (IDE) can also aid in regulating peripheral insulin levels and thus IR signaling [58].

When defining a condition, it is helpful to understand the etiology of that condition. There are many hypotheses regarding the focal initiators of peripheral insulin resistance, including extracellular perturbations such as hyperinsulinemia, glucocorticoids, inflammation, and even aging causing intracellular stress such as oxidative stress, endoplasmic reticulum stress, and dysregulated fatty acid metabolism in metabolic tissues (i.e., skeletal muscle, adipose tissue, and liver) [53,59]. Elevated acute phase proteins like C-reactive peptide and cytokines such as IL-6 and TNF precede the development of insulin resistance [60,61]. Chronically elevated free fatty acids lead to insulin resistance [62] and insulin resistance leads to dysregulated release of free fatty acids from adipose tissue [63], leading to a vicious cycle. Additionally, the adipokine, retinol-binding protein 4 (RBP4), has recently gained attention in its role in peripheral insulin resistance by increasing lipolysis [64]. While these are likely not the only initiators of peripheral insulin resistance, these are the most studied. 

Peripheral insulin resistance can be readily tested by well-accepted clinical methods. Most often this is accomplished by the Homeostatic Model Assessment for Insulin Resistance (HOMA-IR), which involves insulin-stimulated glucose uptake and/or glucose tolerance tests. However, HOMA-IR does not inform us about CNS insulin resistance or even CNS insulin levels because (1) the saturable nature of the BBB transporter means that the relation between blood and CNS levels of insulin are not linear, (2) the CNS is not part of the classic feedback loop controlling insulin release, and (3) as discussed in further detail below, the effect CNS insulin does have on peripheral insulin and glucose levels is opposite to that of peripheral insulin.

Skeletal muscle, fat, and liver are the classic peripheral tissues associated with insulin resistance (Figure 3). The IR signaling pathway described above has been extensively studied in these tissues. IR signaling and multiple post-receptor intracellular events are disrupted in insulin resistance (Figure 3, left side). While this pathway is primarily involved in glucose uptake, insulin can also regulate cell proliferation, gene expression, and suppress hepatic glucose production. Additionally, as noted above, peripheral insulin resistance can occur in select tissues (i.e., hepatic insulin resistance which is defined as the failure of insulin to suppress hepatic glucose production). This also occurs in the skeletal muscle [62]. Often, the tissue selectivity ultimately leads to widespread tissue insulin resistance in the periphery. While this can sometimes extend to the CNS, it is not always the case as discussed above. It will be important to continue exploring this area to determine why peripheral insulin resistance does not always lead to CNS insulin resistance, as it can spread from tissue to tissue in the periphery. This could be related to the role of the BBB in mediating CNS insulin levels or, hypothetically, the ability of the CNS to regulate its own insulin levels [65]. 

Many features of peripheral insulin resistance can have indirect effects on the CNS, being linked back to the BBB. For example, as peripheral insulin resistance is often associated with hyperglycemia, hyperinsulinemia, and increased lipolysis leading to an increase in serum free fatty acids (FFAs), these alterations in serum factors affect the transport and ultimately CNS signaling of critical metabolic hormones [66]. Additionally, as mentioned above, initiators of insulin resistance such as inflammation (elevated cytokine levels) and increased oxidative stress are also known to have effects on BBB function [67,68]. Therefore, it is difficult to determine cause and effect of CNS insulin resistance when associated with peripheral insulin resistance.

Peripheral insulin resistance is a part of the natural aging process. However, each organ and even cells within an organ, ages at different rates [69], making it difficult to compare cell types and organs in aging. Insulin resistance has even been detected in isolated cultured human monocytes. Not only are IR mRNA levels decreased with type 2 diabetes compared to those controls without a history of type 2 diabetes, but the insulin-stimulated IR signaling response is also blunted in those individuals with type 2 diabetes [46]. This study also shows that insulin resistance, as measured by the insulin-stimulated Akt phosphorylation response, correlates with age in both the control group and the type 2 diabetes group. That is, as an individual ages, the ability of the monocyte to respond to insulin declines. This also occurs in the skeletal muscle [70] and appears to be worse in adipose tissue [71]; the liver seems largely spared during aging [71] although there are conflicting reports [72]. Due to the differences in aging signatures in various tissues [69], and these differences in insulin resistance development due to aging, it would be difficult to fully link insulin resistance between all tissues at any given time. 

Determining whether the primary contributor from peripheral insulin resistance (hyperinsulinemia or hyperglycemia) is related to cognitive decline is difficult to answer. On the one hand, in clinical studies, chronically high blood insulin levels correlate with cognitive decline. Elevated peripheral insulin levels are associated with worsened cognitive function [73,74]. Some of the first studies suggest peripheral insulin resistance could be linked to cognitive impairment. However, there are many conditions associated with insulin resistance that are also linked to AD including hyperlipidemia, hyperthyroidism, and hyperglycemia. In a causative study, when blood glucose levels were clamped during acute hyperinsulinemia in healthy, young adults, memory was actually improved [75]. CNS effects due to chronic peripheral resistance versus acute increases in peripheral insulin warrant further comparisons. On the other hand, the loss of peripheral insulin (due to streptozotocin treatment) in a mouse model of AD, the APP/PS1 mouse, associates with increased production of amyloid β (Aβ) in brain, contributing to the pathological features of AD. However, this mouse model of insulin deficiency is also associated with hyperglycemia, increased CNS oxidative stress, pericyte loss, and BBB disruption [76], all of which are implicated in AD, so teasing apart the direct effects of insulin deficiency is difficult. These paradoxical peripheral effects on the CNS again suggest that these two systems may be differentially regulated and that conditions associated with altered peripheral insulin, such as hyperglycemia, dyslipidemia, obesity, sleep apnea, and hypertension, may actually be the contributors to the CNS effect, leaving peripheral insulin as an indirect bystander. To support this, delivering insulin to the CNS can improve memory in both rodent and clinical studies [77]. This highlights the importance of insulin signaling in aging and how preserving the balance of insulin signaling can aid in longevity, with either too little or too much insulin being harmful. 

Ultimately, peripheral insulin resistance is an evolutionarily-conserved mechanism, important in fasting and pregnancy, when the primary fuel source, glucose, needs to be conserved for the brain and developing fetus, respectively. This further supports that there can be a discrepancy in the development of insulin resistance in the periphery compared to in the CNS. While peripheral insulin resistance has been investigated for nearly a century, CNS insulin resistance, particularly in relation to cognition is a newer concept. The role of CNS insulin resistance in energy homeostasis emerged in the 1970′s, while the impact on cognition developed closer to 25 years ago [78,79]. Therefore, we still have much to learn about the impact of insulin in the CNS, specifically related to its role in cognition.

### 3.1. Peripheral Insulin Resistance and COVID-19

Some links between insulin resistance and COVID-19 have been recently investigated. Metabolic factors such as obesity, presence of diabetes mellitus, and hypertension have been associated with a higher likelihood of being infected by SARS-CoV-2 and more severe symptoms of COVID-19. In the study by Denson et al. the presence of metabolic syndrome was associated with an increased risk for admission to the intensive care unit, mechanical ventilation, development of acute respiratory distress syndrome (ARDS), and death [80]. They also found that the likelihood of developing ARDS increased linearly as a function of the number of metabolic syndrome features present. Insulin resistance itself, as measured by the triglyceride and glucose index, is associated with more severe symptoms of COVID-19 and with increased mortality from SARS-CoV-2 [81].

The above studies relate to the effect of diabetes mellitus on the risks associated with COVID-19, but COVID-19 is also a risk for developing diabetes mellitus and insulin resistance. Cromer et al. [82] found that of 1902 patients with COVID-19, 27% had preexisting diabetes and another 4% developed new onset diabetes mellitus. Death rate among the new onset diabetics was exceptionally high at about 17%. Of the survivors with new onset diabetes mellitus, about 56% were still classified as diabetic at a mean follow-up time of 323 days. Montefusco et al. [83] found that about 12% of COVID-19 patients who previously did not have a diagnosis of diabetes mellitus were diagnosable by American Diabetes Association criteria as diabetic, with another 18.5% having transient hyperglycemia. This suggests that COVID-19 can cause both a short-term and a persistent form of diabetes mellitus that can continue long after recovery [82,83]. 

The predominant form of new onset, COVID-19-related diabetes mellitus is characterized by insulin resistance [83,84]. Indeed, the study by Montefusco et al. found elevated insulin and C-peptide levels in both diabetic and euglycemic COVID-19 and post-COVID patients [83]. Similarly, Chen et al. [85] found in non-diabetic COVID-19 patients that markers of insulin resistance were elevated 3–6 months after recovery from COVID-19. These studies show that COVID-19 is associated with the development of insulin resistance, that insulin resistance occurs in both hyperglycemic and euglycemic patients, and that such resistance can persist long after recovery from COVID-19. Several mechanisms for COVID-19-related hyperglycemia and insulin resistance have been proposed and include response to the inflammation of the cytokine storm, elevations in angiotensin II that result from a decrease in ACEII levels, T cell imbalance, and downstream effects of RE1-silencing transcription factor [84,86,87]. Interestingly, metformin, which can alter each of these four mechanisms, has shown some promise in the treatment of COVID-19 in both diabetic and non-diabetic patients [88]. These studies are summarized in Table 1.

It is now well known that some COVID-19 survivors, especially early in the pandemic, develop a form of long COVID, or post-COVID conditions. In a one-year follow up study of nearly 1500 COVID-19 survivors, ages 60 years and older, have long-term cognitive decline [89]. While we do not fully know if SARS-CoV-2 infection can cause AD, there are studies showing AD-like pathology in post-mortem autopsies of those infected that had cognitive impairment [90]. As CNS insulin resistance is associated with cognition, it is currently unknown whether COVID-19 can lead to CNS insulin resistance, as it has been shown for peripheral insulin resistance described above.

### 3.2. Role of apoE Status on Peripheral Insulin Resistance

ApoE genotype has been linked to the development of peripheral insulin resistance, particularly when challenged with risk factors. This in turn might contribute to risk of developing MCI or AD, as being an asymptomatic ε4 carrier was associated with increased phosphorylated and total tau levels in the CSF while the opposite pattern was seen in non-ε4 carriers [91]. However, insulin resistance at midlife was associated with Aβ-positive PET scan in non-ε4 and ε4 carriers, suggesting that ε4 and peripheral insulin resistance might serve as independent risk factors of AD as well [92].

While in humans, E4 carriers are at increased risk to develop type 2 diabetes [93], in the human apoE targeted replacement mice on a high fat diet, fasting glucose and insulin levels were similarly increased in E3 and E4 mice [94]. 

### 3.3. Role of Ethnicity in Central and Peripheral apoE Effects

It is important to recognize the ethnicity differences that cannot be studied in mouse insulin sensitivity, particularly related to *APOE* genotype. In a cross-sectional study involving clinical data for 1025 healthy non-Hispanic White, Hispanic White, East Asian, South Asian and African American individuals, non-Hispanic Whites and African Americans displayed greater insulin sensitivity than East Asians and South Asians [95]. Similar results were seen in earlier smaller studies [96,97,98]. Understanding the ethnicity difference in insulin sensitivity is also important for defining the optimal treatment dose [99]. The risk to develop AD based on *APOE* genotype are ethnicity-dependent [100] and the effects of E4 on resting state functional connectivity (rsFC) in the default mode network (DMN), which is important for cognition, are as well [101]. In non-Hispanic Whites, ε4 carriers have lower rsFC in temporal DMN compared to ε4 non-carriers. In Non-Hispanic Black and Hispanic ε4 carriers, rsFC is slightly higher or similar compared with non-Hispanic White ε4 non-carriers. Thus, ε4 modulates DMN rsFC differently in non-Hispanic Whites compared to non-Hispanic Blacks and Hispanics. Considering the relationship between insulin sensitivity and brain function and the ethnicity-dependent AD risk based on *APOE* genotype, it is likely that peripheral insulin resistance might also reveal ethnicity dependency based on *APOE* genotype. The genetic background might also modulate effects of apoE in the mouse models. Therefore, ongoing efforts involve creating and analyzing AD rodent models of genetically distinct backgrounds is important [102,103]. 

## 4. CNS Insulin Resistance

CNS insulin has a variety of functions (Figure 1), with many likely to still be discovered. CNS insulin can regulate CNS specific molecular signaling functions such as neuronal glucose transporter translocation [104], brain calcium signaling [105], ATP-sensitive potassium channels [106], tau phosphorylation [107], Aβ clearance [108]. Overall brain processes such as reward [109], learning, and memory [110] are also regulated by CNS insulin. Lastly, CNS insulin can affect peripheral processes including blood flow [111], reproduction [112], and peripheral gluconeogenesis [113], leading to regulation of blood glucose levels. As noted above, the idea of insulin resistance within the CNS arose from observations consistent with deficient activity of insulin within the CNS. Broadly speaking, such deficient action could occur because of low levels of insulin within the CNS or because of resistance at the receptor levels (Figure 3, right side). 

Measuring CNS insulin resistance is much more difficult and not as standardized as measuring peripheral insulin resistance. The prevailing ways to measure CNS insulin resistance are to measure CSF to serum insulin levels and post-mortem tissue analysis of phosphorylation events in response to insulin stimulation or total protein levels. However, there is not a CSF to serum insulin threshold specifically defining CNS insulin resistance, as is the case for HOMA-IR. Instead, levels are compared between groups to suggest CNS insulin resistance. Additionally, as noted above, decreased CSF to serum levels relate more to insulin deficiency rather than to classic insulin receptor dysfunction. More recently, clinical CNS imaging is being explored and advanced to investigate CNS insulin resistance in living subjects [114]. A hyperinsulinemic-euglycemic clamp or intranasal insulin is used in concert with imaging to evaluate responses within the CNS using magnetoencephalography (MEG) [115] and FDG-PET [116]. Functional MRI (fMRI) is a new tool that allows for better spatial signal than PET and MEG but has poor temporal resolution. When using hyperinsulinemic clamps though, it is important to consider the BBB and realize insulin transport into the brain is saturable and differentially affected by many conditions [117,118], which could confound interpretations. Another, less used technique to assess CNS insulin resistance is by measuring the ability of insulin-induced potassium ATP channel activation in the hypothalamus to suppress hepatic glucose production [119]. This assay allows for a more easily measured peripheral output but is region-dependent. Lastly, a peripheral biomarker for CNS insulin resistance, enriched neural-derived extracellular vesicles (NEVs) collected from the plasma, recently termed a “liquid biopsy” [120], can be probed for detecting insulin resistance [121]. In this study, brain insulin resistance, defined by protein levels of pIRS-1 present in NEVs, was associated with brain atrophy in AD. A follow-up study using NEVs has shown that enhanced neuronal proximal insulin signaling is associated with preserved brain structure in non-demented aged adults [122]. While these studies show promise for biomarkers of cognitive impairment, whether the biomarkers correlate with post-mortem analysis of classic assays for CNS insulin resistance, including insulin-stimulated IR pathway activation or total IR pathway protein changes, remains to be determined.

Deficient activity of insulin within the CNS can have several causes. One cause of insufficient amounts of insulin could be a decrease in insulin’s inability to enter the CNS; that is, there is a defect in the ability of the BBB to transport insulin into brain (Figure 3). Evidence for this includes a lower CSF to serum ratio in AD subjects than in non-AD subjects [123,124]. The same has been shown for aging. There is an age-dependent decrease in the CSF to serum ratio for insulin in humans [125]. This deficiency was attributed to a role of the BBB. In follow-up studies in aged mice, direct delivery of insulin to the CNS resulted in similar activations of the IR signaling pathway to young mice, whereas peripheral delivery resulted in a blunted signaling event in aged mice compared to young mice [125]. Insulin is transported into the brain by a saturable transporter and evidence shows it is a protein different from that which constitutes the IR [126,127,128,129]. Because it is a different protein, its synthesis and the allosteric regulators which influence its activities could be different from those of the receptor. Plasma triglycerides, for example, increase the rate of insulin transport across the BBB, but triglycerides in the CNS inhibit IR activity [130,131]. 

Another cause of decreased insulin levels in the CNS could be increased clearance of insulin from the CNS, including increased enzymatic degradation. This contrasts to the periphery, where increased insulin clearance from blood would be compensated by increased insulin secretion because of the negative feedback loop between blood glucose and blood insulin levels. Because the CNS is not part of this negative feedback loop, low levels of CNS insulin would not increase blood levels of insulin. However, most studies indicate that IDE, which also degrades Aβ, has decreased activity in AD [132,133]. Therefore, CNS insulin levels and insulin resistance in AD are likely not driven by alterations in IDE activity and insulin degradation.

Another cause of insulin-deficient activity would be resistance at the receptor level (Figure 3). This could be caused by a reduced number of IRs, inhibition of binding between insulin and its receptor, or post-receptor binding issues. IR number has been shown to decrease with aging and even more so in AD [78]. Aβ can interfere with binding of insulin to its receptor [132]. Post-receptor IR signaling deficits occurs in AD subjects who are not diabetic as well as those who are [8,134]. 

Thus, there is evidence that deficient insulin action in AD could have several causes from decreased BBB transport to classic receptor resistance. Because insulin in the CNS is not known to participate in the negative feedback loop, deficient insulin action in the CNS does not self-correct by increasing blood levels of insulin. However, there could be as yet undiscovered mechanisms by which CNS insulin could act to maintain its levels as blood levels fluctuate. In the next section, we consider mechanisms by which CNS insulin might affect the regulation of its own levels.

While the etiology of peripheral insulin resistance has been investigated, whether similar initiators are responsible for CNS insulin resistance are unknown, but are a current area of active research. It is important to distinguish whether these initiators are truly initiators of CNS insulin resistance or arise due to the development of CNS insulin resistance. This discrepancy can be hard to parse out and therefore associations are simply made. As in the periphery, inflammation [135] and aging [9] have also been shown to be associated with CNS insulin resistance. Additionally, mitochondrial dysfunction and oxidative stress are linked to CNS insulin resistance [67]. One driving factor in the development of CNS insulin resistance is inhibition of biliverdin reductase-A (BVR-A), an important upstream regulator of the IR signaling pathway [136]. BVR-A is inhibited by oxidative stress and this inhibition is exacerbated in an animal model of AD, leading to increased AD pathology [136,137]. BVR-A protein levels are also decreased in peripheral blood mononuclear cells in patients with type 2 diabetes [138]. Teasing apart causal versus correlative initiators of CNS insulin resistance in humans will be difficult until a standardized CNS insulin resistance test, like the HOMA-IR for the periphery, has been defined.

As mentioned earlier in the Peripheral Insulin Resistance section, there can be tissue selectivity when it comes to peripheral insulin resistance. While much of this selectivity is often diminished over time and widespread insulin resistance ensues, whether a similar path occurs in the CNS is unknown. Instead of looking at tissue selectivity in the CNS which is primarily comprised of brain tissue, it is worth breaking down the brain into cell type. First, whether there is CNS cell specificity when it comes to insulin resistance has not been tested. Especially in the context of AD, aging, and age-related cognitive decline, it will be important to understand whether a particular CNS cell type drives insulin resistance in the brain. That is, does CNS insulin resistance begin in or primarily affect one cell type or are all CNS cell types equally susceptible to the development of insulin resistance. The sensitivity of pericytes to peripheral insulin resistance [76] suggests this latter point is not the case. Some genetic and targeted knock-down models have begun to explore cell-specific contributions to CNS insulin resistance [65] but looking at the various cell types in the same sample have not been explored. The use of single-cell technology will allow us to begin to answer this question and some suggestions are already present showing reduced mRNA expression in AD (Figure 2D). However, instead of solely looking at mRNA expression of the IR as detailed above, it will be important to analyze changes in IR protein level, isoform ratio, and phosphorylation events in the brain. Additionally, knowing whether the development of insulin resistance in one cell type then spreads to other cell types will help combat this dysfunction. If there are cell types that are more resistant to the development of insulin resistance, this will help highlight protective pathways. Additionally, regarding CNS cell-type specificity, whether insulin resistance in the neuron or brain endothelial cell means the same thing in the astrocyte is unclear. The insulin-stimulated glucose transporter, GLUT4, is expressed in the CNS and mRNA levels are greater in the astrocytes than other cell types [48]. Therefore, insulin resistance in astrocytes could be related to and impact glucose uptake by this specific cell type, whereas this may not be the case for neurons. 

While we have focused on aging and AD in this section related to CNS insulin resistance, there are also studies investigating the impact of CNS insulin resistance in other conditions, including neuropsychiatric disorders [139,140] and conditions involving alterations in peripheral metabolism [141]. CNS insulin resistance does not just impact the CNS. There is cross talk between the brain and periphery, as recently reviewed [142]. We do not have the space to fully cover the CNS contributions to peripheral metabolism. However, we do want to point out that intranasal insulin can improve cerebral blood flow (CBF) in healthy volunteers and those with type 2 diabetes [143,144]. Enhanced CBF is associated with improved cognition and declines with age [145]. While AD patients with type 2 diabetes have lower cognitive function at baseline, the rate of decline in those with type 2 diabetes did not differ from those without [146]. This suggests that the progression of AD cognitive impairment can be separated from the peripheral effects of type 2 diabetes. Additionally, dietary restriction and lifestyle interventions can restore whole body insulin sensitivity in people with type 2 diabetes [114]. Acute exercise can increase insulin transport across the mouse BBB but the effects on CNS insulin signaling in healthy, young mice were less clear [118]. Follow-up studies are warranted to fully evaluate the impact of exercise on CNS insulin signaling in AD. While improvement in peripheral insulin sensitivity can lead to improvements in brain insulin sensitivity [147], it is unclear whether the opposite is true as well. 

### Impact of ApoE Status on CNS Insulin Resistance

Insulin signaling in the brain can be differentially regulated by distinct apoE isoforms [148]. Consistent with this notion, apoE isoforms modulate the beneficial effects of intranasal insulin treatment on cognition in AD patients, particularly in women [149,150]. Additionally, conditional deletion of the major apoE receptor, LDLR-related protein 1 (LRP1), in the forebrain, impaired brain insulin signaling [151]. Studies in human apoE targeted replacement mice also support that apoE isoforms differentially regulate insulin signaling. In E4 mice, CNS insulin signaling is impaired [152]. This effect is more profound when the mice receive a high-fat diet. In vitro studies with primary neuronal cultures indicate that E4 interacts with the IR and by trapping it in endosomes, impairs trafficking causing impaired insulin signaling and insulin-stimulated mitochondrial respiration and glycolysis [152]. E4 and peripheral insulin resistance also interact to impair cognition and alter the hippocampal epigenome and metabolome [94]. While both E3 and E4 mice fed a high-fat diet show impairments in peripheral metabolism and cognition, impairments in hippocampal-dependent spatial learning and memory are more profound in E4 mice. Using genome-wide measures of DNA hydroxymethylation with comprehensive untargeted metabolomics, novel alterations in purine metabolism, glutamate metabolism, and the pentose phosphate pathway were identified. Differential effects due to apoE isoforms in response to a peripherally administered glucose bolus are also observed in E3 and E4 mice on a high-fat diet. E4, but not E3, mice show an acute improvement in cognition and cerebrovascular response [153]. For a review about apoE and cerebral insulin, please see [15]. The apoE isoform-dependent effects start early in life and do not require an environmental challenge like a high-fat diet. In young E3 and E4 mice, levels of insulin binding the vasculature at the BBB are different due to apoE isoform and sex [154]. In aged E3 and E4 mice, some measures of insulin function also support apoE isoform-dependent effects [155]. Insulin BBB transport in aged E3 and E4 mice on a low-fat and high-fat diet show genotype specific effects, being more profound in females. Striatal and hippocampal ^1^^25^I-insulin transport was absent in E4 mice but present in E3 mice. In contrast, hypothalamic ^125^I-insulin transport is decreased in E3 mice on a high-fat diet while increased in E4 mice on a high-fat diet. These data suggest an important apoE isoform-dependent regulation of the insulin transporter at the BBB. Regulation of this transport system by apoE could be an underlying cause of some of the apoE isoform CNS effects in AD.

## 5. Contributions of the Periphery to CNS Insulin Actions

It is thought that the majority of CNS insulin is derived from blood. There is evidence of limited insulin production in some areas of the CNS [156]. Perhaps the limited CNS insulin production can have immediate effects in the local milieu, which has recently been shown in drosophila [157]. However, if these areas affect the larger picture of CNS insulin, much of what is currently believed would have to be reconsidered. However, current thought maintains that the CNS is ultimately dependent on the pancreas for its insulin. 

The transport of insulin across the BBB involves a saturable system [6,126]. This means that although higher blood levels of insulin result in higher levels of insulin within the CNS, the relative amounts of insulin entering the CNS diminish as blood levels of insulin increase. Thus, at higher blood levels of insulin, the CSF to serum insulin ratio decreases and at lower blood levels of insulin, the CSF to serum ratio increases. 

The insulin transporter is known to be regulated as its rate of transport is influenced by several conditions and substances, including exercise, obesity, starvation, inflammation, triglycerides, cholecystokinin, and nitric oxide [118,130,158,159,160,161]. One mechanism by which peripheral insulin resistance could result in CNS insulin resistance involves hypertriglyceridemia, the major dyslipidemia associated with insulin resistance and diabetes mellitus. Triglycerides can cross the BBB and once in the brain, they induce hypothalamic insulin resistance [131].

One theoretical way that the CNS could control its supply of insulin would be if the transporter at the BBB is regulated by actions within the brain. Other BBB transporters, such as that for leptin, are known to be influenced by astrocytic input [162]. If the BBB insulin transporter is similarly regulated by inputs from astrocytes, microglia, or pericytes or by neuroimmune events, this could be a major mechanism by which the brain controls its levels of insulin. This is an area of active research both by our group [65,117] and others [139,163,164]. Further understanding what regulates insulin BBB transport could aid in restoring CNS insulin levels in deficient states such as aging and AD.

### ApoE-Related Serum Factors Contributing to CNS Insulin Action

There is a clear link between apoE isoform expression, the insulin-related protein, α-klotho, insulin resistance, and cognition. Alpha klotho in blood might modulate CNS insulin action. In the pancreas, α-klotho enhances glucose-induced insulin secretion [165]. Alpha klotho suppresses insulin signaling [166]. Mouse and human α-klotho share 80% homology [8]. Heterozygote carriers of the KL-VS α-klotho variant have higher α-klotho serum levels [6,9] and show better global cognition [6]. Carrying one copy of the KL-VS α-klotho variant predicted better cognitive outcomes [6] and was also associated with a larger right dorsolateral prefrontal cortex volume [12]. In healthy older adults, higher serum α-klotho levels were associated with greater intrinsic connectivity within the fronto-parietal network, the default mode network, and other functional networks vulnerable to AD-related cognitive decline [9]. Alpha klotho in brain can also affect CNS insulin action. Alpha-klotho levels are high in the CSF [1,2,3] and related to insulin signaling, cognition, and lifespan in mice [1,3,4,5,6]. Alpha-klotho protein levels in the CSF are lower in women than men, lower in older adults with AD than age-matched controls, and decline with age [3]. Alpha klotho levels in the CSF are lower in obese/overweight than lean people and central administration of alpha klotho increases energy expenditure [167]. Whether apoE isoform modulates the effects of α-klotho is not clear. High α-klotho levels were shown to especially benefit apoE4 carriers [8,16]. However, in people between 39 and 90 years of age, serum and CSF levels of α-klotho are positively correlated and predict scores on both the Mini-Mental State Examination test and the Clinical Dementia Rating regardless of sex or *APOE* genotype [168]. There is a robust positive relationship between α-klotho and apoE levels in the amygdala in irradiated non-human primates [18]. Klotho is also important in reducing Aβ pathology. Klotho overexpression improves Aβ clearance and cognition in APP/PS1 mice [169]. Insulin in turn positively regulates α-klotho levels. In cultured hippocampal neurons, insulin increases neuronal α-klotho levels [170]. Subsequently, this enhances formation and release of lactate from astrocytes that can be used as metabolic substrate. Acting in a negative feedback loop, soluble klotho then inhibits insulin signaling [171]. Consistent with this notion, reductions in insulin signaling suppresses aging-like phenotypes in klotho deficient mice [172]. As indicated before, this also highlights that either too little or too much insulin in harmful for healthy aging.

## 6. Influences of CNS Insulin on Peripheral Action

**Figure 4:**
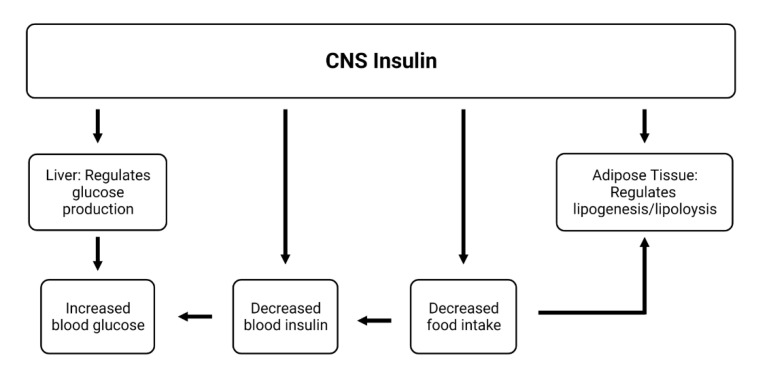
Influence of CNS insulin on the periphery. CNS insulin has been shown to have many impacts on peripheral metabolism including increasing blood glucose, decreasing blood insulin, and decreasing food intake. Some of these impacts may be regulated by the sympathetic effect of insulin on liver glucose production and/or adipose tissue regulation of lipogenesis and lipolysis. Many of these influences impact insulin BBB transport, such as serum triglyceride and insulin level, which ultimately affects CNS insulin level.

Evidence suggests that there are influences of CNS insulin on events in the periphery. The predominant currently known effects of CNS insulin on peripheral action relate to metabolic changes. Insulin injected into the lateral ventricle of the brain has the opposite effects of insulin injected peripherally, increasing blood levels of glucose, decreasing food intake, and decreasing blood levels of insulin (Figure 4) [173,174,175]. This suggests that although CNS insulin does not participate in the classic negative feedback loop of glucose/insulin in the periphery, it is linked to this system by some other mechanism. One such way is that CNS insulin by way of vagal efferent sympathetic outflow [176] regulates liver production of glucose [119,177]. Brain insulin also influences lipolysis and lipogenesis in adipose tissue [178]. Changes in serum factors induced by these processes have been shown to affect insulin transport into the brain, which could complete this cycle.

Features of AD can induce peripheral metabolic deregulation in mice [179]. Specifically, injection of amyloid beta oligomers into the CNS of healthy mice triggers peripheral glucose intolerance. Systemic injections do not elicit the same effect, suggesting the initial effect lies within the CNS. Hypothalamic inflammation is ultimately revealed as the primary culprit in the CNS amyloid beta oligomer induction of peripheral glucose deregulation. We are not aware of any human studies that have explored the development of type 2 diabetes in patients with AD. 

### Impact of CNS ApoE on the Periphery

As described earlier, mice lacking murine apoE and only expressing human apoE3 or apoE4 in brain show a similar increase in plasma corticosterone following restraint stress as mice lacking murine apoE that do not express human apoE in brain [29], despite apoE isoform-dependent changes in hippocampal neuropathology [31]. Based on this result, one might conclude that CNS apoE might have no effect on the periphery. However, with the increased recognition of the important role of the bi-directional gut-liver-brain axis, CNS apoE could, at least, indirectly affect the periphery. The gut microbiome can communicate with the CNS via the gut–brain axis and affect behavioral phenotypes [180,181,182,183,184,185,186]. ApoE could be important in modulating the gut-liver-brain axis based on the specific gut microbiome profiles in humans and human apoE targeted replacement mice [187]. Using a mouse model of AD (the hAPP knock-in mice, *App^NL-G-F^*), behavioral and cognitive performance is associated with the gut microbiome and the APP genotype modulates this association [188]. The gut microbiome is also linked to hippocampal DNA methylation in these mice in the promoter region of the *Apoe* gene, with more methylation in the hippocampus of *App^NL-G-F^* than wild type mice. To further support this role for apoE in the modulation of the gut-liver-brain axis was revealed in a study involving inoculation of germ-free recipient mice with stool from 6-month-old *App^NL-G-F^* mice or *App^NL-G-F^* mice crossed with human apoE4 targeted replacement mice (*App^NL-G-F/E4^*) [189]. Recipients of *App^NL-G-F^*, but not *App^NL-G-F/E4^* donor mice, had cortical insoluble Aβ40 levels that positively correlated with activity levels on the first and second day of open field testing, implicating a role of E4. Final evidence to support the link between the gut microbiome and apoE is that apoE binds to triggering receptor expressed on myeloid cells 2 (TREM2) [190]. TREM2 in the gut [191] and in microglia [192,193] has been implicated in AD-related neuropathology [191,194]. Thus, specific microbes may impact AD-relevant phenotypes via epigenetic changes in AD-susceptibility genes in the hippocampus and by inducing neuroinflammation via the gut–brain axis in an apoE isoform-dependent fashion.

## 7. Conclusions and Remaining Questions

In this review, we have tried to highlight similarities and differences between peripheral and central insulin resistance and whether one can lead to the other. The classic definition of insulin resistance may not be the same between the two sites. We have tried to demonstrate how insulin resistance can occur in the periphery and the brain. We discussed ways to measure insulin resistance, both in living humans and post-mortem samples. We also discussed how CNS insulin resistance may often be a case of insulin deficiency, where delivery of insulin to the CNS overcomes any signaling deficiencies. This is often not an issue in the periphery except in type 1 diabetes when pancreatic insulin secretion is low or absent. On the other hand, there are clear examples of true insulin resistance both in the periphery and CNS, defined by the blunted action of insulin on the IR signaling pathway, most commonly measured by phosphorylation events following insulin stimulation. Despite our extensive knowledge behind peripheral and central insulin resistance, some questions remain. To what extent is the connection between peripheral insulin resistance and AD due to peripheral vascular events (such as hypertension, multi-infarct dementia, and stroke) rather than events within the CNS (i.e., CNS insulin resistance)? To what extent are other conditions that accompany peripheral insulin resistance (such as hypertension, obesity, hyperlipidemia, and hyperglycemia) detrimental to cognition? What role does apoE genotype really play in both peripheral and/or central insulin resistance? What are the downstream effects of CNS insulin resistance and what are the mechanisms by which CNS insulin influences cognition? Although type 2 diabetes might contribute to AD, it is not necessarily sufficient to trigger it. The fact that type 1 diabetes does not have the effect of type 2 diabetes highlights the role of insulin sensitivity versus presence/absence of insulin. Future efforts are warranted to assess CNS insulin sensitivity in cognitive disorders other than type 2 diabetes and AD as well.

Insulin resistance is clearly associated with aging and AD, whether predominating in the CNS, in the periphery, or both. Therefore, further understanding this relationship and working to define the dysregulation of CNS insulin signaling more clearly, will allow us to more directly target this feature present in aging and AD. While peripheral and central insulin resistance have connections, it is a far, far better thing to consider them as independent, interacting processes.

## Figures and Tables

**Table 1:** Peripheral insulin resistance and COVID-19.

Relationship	Condition/Treatment	Patient Numbers *	Mean Age	Study Type	Date of Study	Inclusion Criteria	Population %	Main Findings	COVID-19 Mortality	Notes	Reference
Diabetes worsenes COVID-19 outcome	Metabolic Syndrome	46,441	61.2 ± 17.8 years old (SD)	Retrospective	15 February 2020 to 18 February 2021	Completed discharge status	17.5% had metabolic syndrome	Increased risk of ICU admission, invasive mechanical ventilation, ARDS, and mortality; increased ICU and hospital LOS	Increased	MS defined as 3 or more conditions: obesity, prediabetes or diabetes, hypertension, and dyslipidemia)	[80]
	Triglyceride and Glucose Index (TyG)	151	59.5 ± 15.9 years old (SD)	Retrospective	12 January 2020 to 13 Febreuary 2020	Completed medical records and follow-up data	25.8% had diabetes	TyG index levels were significantly higher in the severe cases and death group	Increased	TyG: marker of insulin resistance	[81]
	Diabetes	1902	64 years old	Retrospective	1 March 2020 to 27 September 2020	COVID-19	31.2% had diabetes	36% admitted to the ICU	19% of those with diabetes died		[82]
COVID-19 increases risk for developing diabetes	Newly Diagnosed Diabetes Mellitus (NDDM)	594	54.1 years old	Retrospective, with follow-up observations	1 March 2020 to 27 September 2020	COVID-19 and Diabetes	13% had NDDM	Younger age in NDDM; NDDM had lower glucose levels but worsened COVID-19 (increased LOS, ICU admission); 56% still classified as DM at mean follow up of 323 days	No effect of NDDM	NDDM defined as fasting blood glucose >125–140 mg/dL or any glucose >140–180 mg/dL during admission	[82]
	Development of diabetes	551	61 ± 0.7 years old (SEM)	Retrospective	1 February 2020 to 15 May 2020	No pre-existing diabetes	46% hyperglycemic; 27% normoglycemic	12% had new classification of diabetes; and 18.5% had transient hyperglycemia; DM incread LOS; Glycemic abnormalities persisted for at least 2 months after resolved COVID-19	DM increased		[83]
	COVID-19 induction of diabetes	124	Non-severe COVID: 36.6 ± 15.8 years old; Severe COVID: 59.0 ± 13.9 years old (SEM)	Retrospective	22 January 2020 to 7 April 2020	No pre-existing diabetes	25.8% had metabolic-related diseases	COVID-19 increased blood glucose and insulin levels compared to controls and persisted after virus elimination	Did not investigate	Compared to 30 non-COVID controls; looked into mechanism	[84]
	COVID-19 induction of diabetes	64	44.3 ± 13.5 years old (SD)	Prospective	17 January 2020 to 9 February 2020 (initial cohort)	No pre-existing diabetes	84% had mild COVID; 15.6% had severe COVID	C-peptide and TyG indices increased with decreased fasting glucose levels up to 6 months post discharge		Followed patients at 3 and 6 months post hospital discharge	[85]
Treating COVID-19 with Diabetes Drugs	Metformin treatment	6659 in the 3 observational studies		Systematic Review	Up to 30 July 2020	English publications selected by 3 independent reviewers	9 out of 14 articles	Positive benefit of metformin treatment in COVID-19 w/or w/o diabetes	2/3 studies showed decreased mortality	Keywords used: COVID-19, SARS-CoV-2, 2019-nCoV, metformin, and antidiabetic drug	[88]

* Patient numbers are numbers of admitted COVID-19 patients unless specified as specific population.

## Data Availability

Data to generate Figure 2 was publicly accessed as referenced in the Figure legend.
